# Supplementary material for: The Construction and Exploration of a Comprehensive MicroRNA Centered Regulatory Network in Foxtail Millet (Setaria italica L.)
Source: Front Plant Sci. 2022 May 6;13:848474. doi: 10.3389/fpls.2022.848474 (PMC9121102; doi:10.3389/fpls.2022.848474)
Supplement: Supplementary file 6 [file Data_Sheet_6.zip › Sit-miRNovel2_Seita.3G277000.1_234_TPlot.pdf]

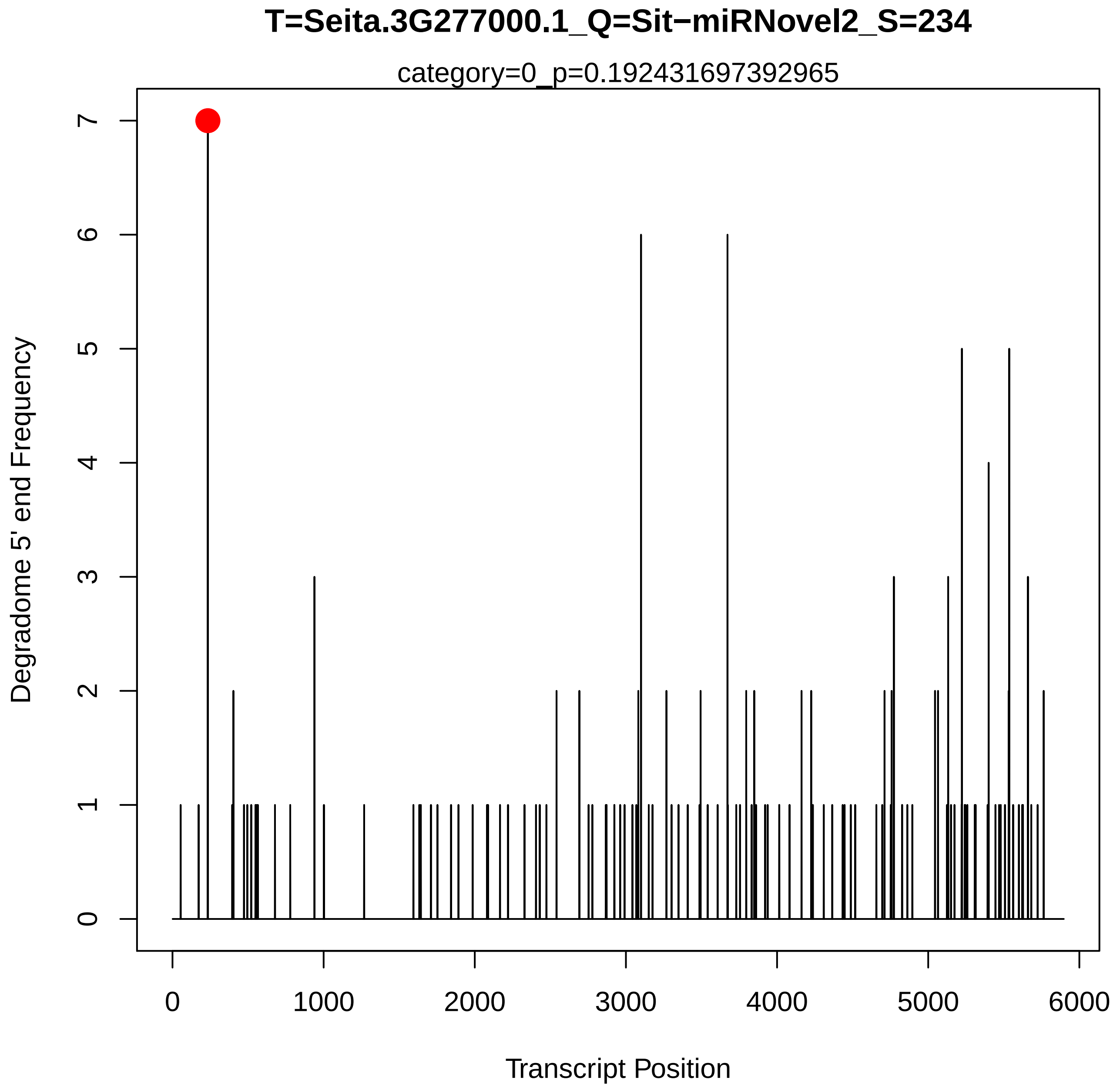

T=Seita.3G277000.1_Q=Sit−miRNovel2_S=234
category=0_p=0.192431697392965
Degradome 5' end Frequency
0
1
2
3
4
5
6
7
0
1000
2000
3000
4000
5000
6000
Transcript Position
